# Discovery of New Chemical Tools against *Leishmania amazonensis* via the MMV Pathogen Box

**DOI:** 10.3390/ph14121219

**Published:** 2021-11-24

**Authors:** Atteneri López-Arencibia, Ines Sifaoui, María Reyes-Batlle, Carlos J. Bethencourt-Estrella, Desirée San Nicolás-Hernández, Jacob Lorenzo-Morales, José E. Piñero

**Affiliations:** 1 Instituto Universitario de Enfermedades Tropicales y Salud Pública de Canarias, Campus de Anchieta, Universidad de La Laguna, Avda. Astrofísico Fco. Sánchez, S/N, 38203 La Laguna, Spain; isifaoui@ull.edu.es (I.S.); mreyesba@ull.edu.es (M.R.-B.); cbethene@ull.edu.es (C.J.B.-E.); dsannico@ull.edu.es (D.S.N.-H.); jpinero@ull.edu.es (J.E.P.); 2 Departamento de Obstetricia y Ginecología, Pediatría, Medicina Preventiva y Salud Pública, Toxicología, Medicina Legal y Forense y Parasitología, Universidad de La Laguna, 38203 La Laguna, Spain; 3 Red de Investigación Colaborativa en Enfermedades Tropicales (RICET), 28006 Madrid, Spain; 4 Consorcio Centro de Investigacion Biomedica en Red M.P. (CIBER) de Enfermedades Infecciosas, Instituto de Salud Carlos III, 28006 Madrid, Spain

**Keywords:** *Leishmania amazonensis*, screening, chemotherapy, Pathogen Box, hits

## Abstract

The protozoan parasite *Leishmania* causes a spectrum of diseases and there are over 1 million infections each year. Current treatments are toxic, expensive, and difficult to administer, and resistance to them is emerging. In this study, we screened the antileishmanial activity of the Pathogen Box compounds from the Medicine for Malaria Venture against *Leishmania amazonensis*, and compared their structures and cytotoxicity. The compounds MMV676388 (3), MMV690103 (5), MMV022029 (7), MMV022478 (9) and MMV021013 (10) exerted a significant dose-dependent inhibition effect on the proliferation of *L. amazonensis* promastigotes and intracellular amastigotes. Moreover, studies on the mechanism of cell death showed that compounds 3 and 5 induced an apoptotic process while the compounds 7, 9 and 10 seem to induce an autophagic mechanism. The present findings underline the potential of these five molecules as novel therapeutic leishmanicidal agents.

## 1. Introduction

Protozoa belonging to the *Leishmania* genus are the causative agents of a complex disease, endemic to tropical and subtropical areas, known as leishmaniasis [1]. Leishmaniasis has been recognized as a growing global health problem by the WHO; it is fourth place for morbidity and the second for mortality rates amongst all tropical diseases [2]. Based on species type and host immune system responses, leishmaniasis takes three basic clinical forms: cutaneous, mucocutaneous, and visceral [3]; first-line treatment involves pentavalent antimonial formulations and sodium stibogluconate. Alternatives include miltefosine, amphotericin B, paromomycin, and pentamidine. However, these molecules feature elevated costs, high toxicity, and difficulties associated with parenteral administration and drug resistance [4,5].

Treatment failure is an important problem to consider because it can occur due to numerous factors in the host (immunity or nutritional status), the parasite (drug resistances, whether the parasite resides in tissues not accessible to drugs), and the environment (with global warming contributing to the expansion of the disease to new geographical areas). Therefore, the emergence of resistant strains, together with the problems mentioned above, is an added problem for the development of new treatments, since, as several studies have shown, drug resistance associated with the current available treatments has started to emerge, even in combination therapies. The development of the next generation of leishmaniasis treatment will be a new challenge for WHO [6].

Consequently, the development of innovative, effective, and safe antileishmanial molecules with reduced side effects is a priority for researchers. For all these reasons, the scientific community is attempting to obtain novel or known molecules with leishmanicidal activity; the Medicines for Malaria Venture (MMV, Switzerland) Pathogen Box library kindly provide 400 diverse compounds, drug-like molecules active against neglected diseases of interest. To this end, this study reports on the screening and study of the MMV Pathogen Box molecules.

Regarding the characteristics of the programmed cell death (PCD) or apoptosis-like process, many morphological features can be observed in the parasite, such as cytoplasm condensation, decreased cell volume, reduced mitochondrial membrane potential, chromatin condensation or DNA fragmentation, among others [7,8]. In apoptotic cells, the plasma membrane loses phospholipid asymmetry, and phosphatidylserine (PS) is externalized, becoming exposed to the extracellular environment, allowing recognition by phagocytic cells such as macrophages [9].

Many researchers have obtained good results in previous studies with the MMV Pathogen Box, where they studied the biological activity against different parasites. For example, potent activity was seen in sixteen of the molecules against *Acanthamoeba castellanii* Neff [10], twenty-three compounds with good activity against of *Balamuthia mandrillaris* were identified [11], fifteen compounds that exhibit activity against *Plasmodium falciparum* were observed [12], and eight compounds with a strong selectivity index against *Toxoplasma gondii* were noted [13]. Other authors have studied the effect on trematoda such as *Fasciola hepatica*, discovering seven compounds with activity against both metacercaria and adult stages [14].

## 2. Results

**Figure 1:**
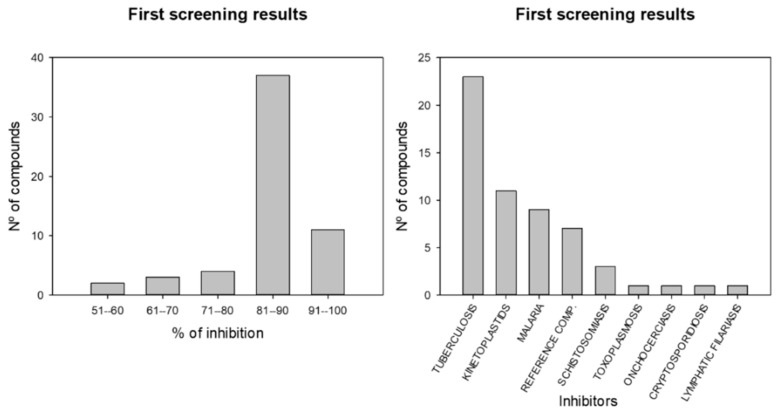
Results of the first screening from the 400 molecules. Data represent the molecules that induce an inhibition of the parasite of higher than 51% at 10 µM. First graph englobes molecules by their percentage of inhibition against *L. amazonensis*, and second graph divides molecules by their biological activity.

After the first screening of the Pathogen Box, we selected the compounds that displayed leishmanicidal activity against promastigotes of *L. amazonensis* with an inhibition of 51% or higher at 10 µM (Figure 1). We discovered that 57 of the 400 compounds achieved this threshold; most of them induced an inhibition between 81–90% (37 compounds) followed by a group that exhibited an inhibition higher than 91% (10 compounds). Furthermore, most of these compounds belong to the group of tuberculosis-inhibitor compounds (23 compounds), followed by kinetoplastids (11 compounds) and malarial inhibitors (9 compounds).

After preliminary screening, we selected the group of compounds with a percentage of inhibition against the parasite higher than 91% at 10 µM. This new group consisted of ten compounds, five of which belonged to tuberculosis inhibitors, three to anti-malarials, one to anti-kinetoplastids, and one to reference drugs. This reference drug is Bedaquiline (1), a known anti-tuberculosis drug. Subsequently, we evaluated the inhibitory effect of the ten molecules against promastigote and amastigote forms of *L. amazonensis*; the results are shown in Table 1. The results for compounds 5 and 9 revealed IC50 values against promastigotes of 0.59 and 0.77 µM respectively, the most active compounds, followed by 3, 4, 7, and 10 with IC_50_ values below 2 µM. The molecular structures of the eleven compounds are related in Table 2.

In relation to the cytotoxic effect against the murine macrophages, half of the selected compounds presented a CC_50_ higher than 10 µM. The others presented results near 8 µM.

Our susceptibility values against miltefosine coincided with those obtained by other authors, such as Alonso et al. [15] with IC_50_ values of 7.8 and 1.8 µM, or those of Trinconi et al. [16], with IC_50_ values of 16.8 and 2.6 µM, both against intracellular promastigotes and amastigotes, respectively. All the compounds displayed activity that was equal or superior to miltefosine against promastigotes; however, when looking at the results of intracellular amastigotes, only four of them improved the results of miltefosine.

Our results demonstrated the good activity of the compounds 5 and 10 against *L. amazonensis* intracellular amastigotes, with IC_50_ values of 1.25 and 1.33 μM, respectively. The other five compounds showed moderate activity, with IC_50_ values between 2.50 and 8.52 µM. Furthermore, three of them either lost their activity or displayed activity higher than 10 µM (results on Table 3).

It is important to mention that the ten compounds achieved Lipinski’s rule of five (data not shown, available in CHEMBL database, https://www.ebi.ac.uk/chembl/ (accessed on 15 May 2021), which determines whether a compound offers chemical and physical properties that would make it susceptible to becoming an orally active drug in humans.

Putting these results all together, we decided to continue with the analysis of physiological events induced by just some of the selected molecules. The selection was based on their selectivity indexes, and the selected compounds were: 3, 5, 7, 9, and 10.

### 2.1. Characterization of the Compounds

Characterization of the MV Pathogen Box compounds was accomplished by TOF-MS. The mass of all the compounds extracted from the MS spectra agreed with the calculated mass, as indicated below (see also Supplementary Materials):

Compound 1 (C_33_H_22_BrNO_2_): calculated mass: 553.2, and observed m/z fragment ([M+Na]^+^): 576.2

Compound 2 (C_16_H_10_ClN_2_O_3_S): calculated mass: 345.0, and observed m/z fragment ([M+Na]^+^): 368.4

Compound 3 (C_15_H_14_N_4_O_3_S): calculated mass: 330.1, and observed m/z fragment ([M+Na]^+^): 353.0

Compound 4 (C_8_H_10_N_3_O_4_S_2_): calculated mass: 276.0, and observed m/z fragment ([M+Na]^+^): 298.0

Compound 5 (C_19_H_23_N_7_): calculated mass: 349.2, and observed m/z fragment ([M+Na]^+^): 372.2

Compound 6 (C_20_H_18_N_4_O): calculated mass: 330.1, and observed m/z fragment ([M-H]^-^): 329.1

Compound 7 (C_26_H_31_N_3_O_2_S): calculated mass: 449.2, and observed m/z fragment ([M-H]^-^): 448.3

Compound 8 (C_24_H_26_F_4_N_8_O_2_): calculated mass: 534.2, and observed m/z fragment ([M-H]^-^): 533.2

Compound 9 (C_23_H_22_ClN_6_O): calculated mass: 433.2, and observed m/z fragment ([M-H]^-^): 432.3

Compound 10 (C_18_H_22_N_4_): calculated mass: 294.2, and observed m/z fragment ([M+Na]^+^): 317.2

### 2.2. Mitochondrial Function

**Figure 2:**
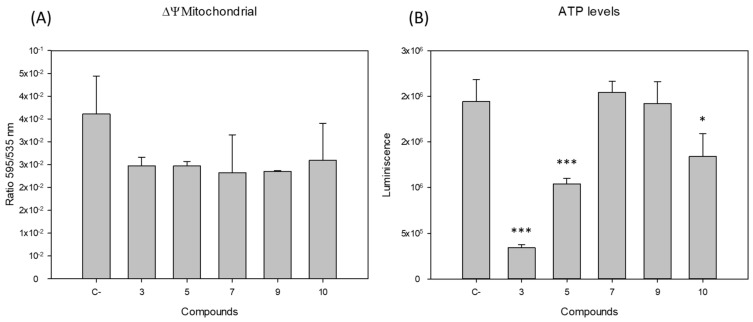
(**A**) Changes in the mitochondrial membrane potential (ΔΨm) and (**B**) ATP levels of *Leishmania amazonensis* promastigotes after 24 h of incubation with the IC_90_ of the compounds. C-: Negative control (not treated parasites). Error bars represent the standard deviation (SD). Each data point indicates the mean of the results of three measurements (***) *p* < 0.001, (*) *p* < 0.01.

Regarding the mitochondrial functioning, we observed that all the tested compounds slightly decreased the mitochondrial membrane potential of the promastigotes after 24 h of incubation, but with no statistical significance (see Figure 2). However, on the other hand, the ATP levels of the parasites after incubation with the IC_90_ of the compounds resulted in a strong decrease in ATP production when incubated with compounds 3 and 5, and a less pronounced decrease with 10.

### 2.3. Oxidative Stress

**Figure 3:**
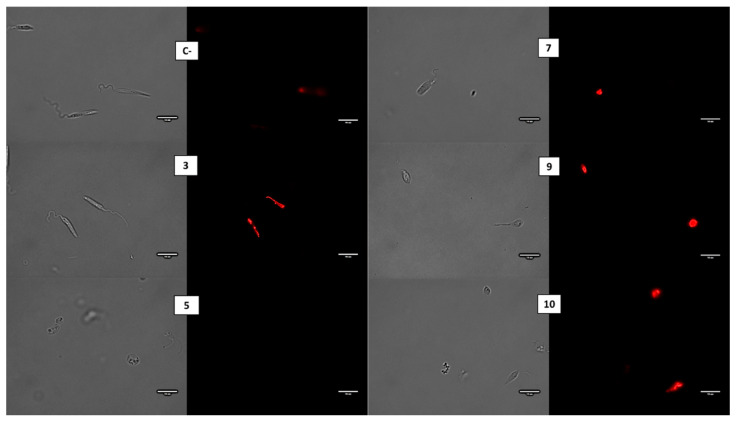
CellROX Deep Red staining. Results after 24 h of incubation of *L. amazonensis* promastigotes with the IC_90_ of compound. C-: Control. Images were captured using an EVOS FL Cell Imaging system (Thermo Fisher Scientific) (100×). Scale: 10 µM.

CellROX Deep Red was used to assess the presence of reactive oxygen species (ROS) in the promastigotes of *L. amazonensis* exposed to the compounds, corroborated by visual fluorescence. As expected, the untreated parasites (negative controls) did not display any increase in CellROX fluorescence compared to the treated *L. amazonensis* parasites with the IC_90_ of the five compounds for 24 h, which increased the visual fluorescence of CellROX Deep Red compared to the untreated cells when observed under the microscope. In Figure 3, we could observe that all the analyzed compounds caused a ROS accumulation inside the parasite after 24h of treatment, except for compound 5, where the parasites appeared to be too damaged to contain the dye in their cellular interior.

### 2.4. Membrane Alterations

**Figure 4:**
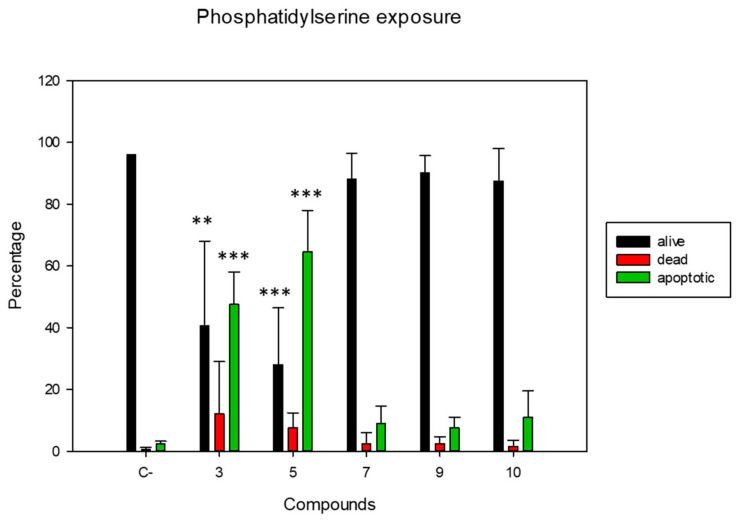
Results of the phosphatidylserine exposure after 48 h of incubation with the IC_90_ of the molecules. Images were captured using a Tali image-based cytometer. C-: Negative control (not treated parasites). Error bars represent the standard deviation (SD). Each data point indicates the mean of the results of three measurements (***) *p* < 0.001, (**) *p* < 0.01.

The cytoplasmic membrane is one of the organelles that first begins to respond to external and internal stimuli. In this respect, when we incubated the different compounds against *L. amazonensis* promastigotes at IC_90_ for 24 h, we observed changes in the arrangement of phosphatidylserine. Normally, this phospholipid is exposed on the inner side of the membrane, but during apoptosis it moves to the outer side of the membrane. Using annexin v and propidium iodide, we separated the population into live, dead, and apoptotic according to their staining. Figure 4 displays the results obtained, which demonstrates that the compounds 3 and 5 caused a strong translocation of phosphatidylserine in the parasite population, higher than 50%. On the other hand, the rest of the compounds also stimulated translocation, although in a smaller part of the population, slightly less than 20%. An increase in the dead population was also observed in a higher percentage of the parasite population when incubated with the compounds 3 and 5, compared to the rest of the treatments.

**Figure 5:**
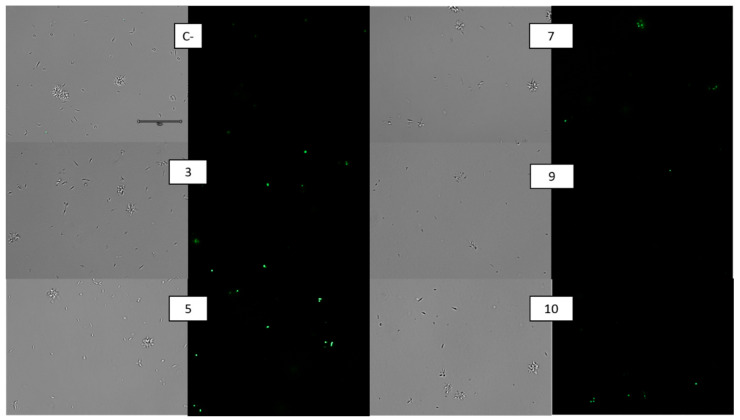
SytoxGreen staining. Results after 24 h of incubation of *L. amazonensis* promastigotes with the IC_90_ of compound. C-: Control. Images were captured using an EVOS FL Cell Imaging system (Thermo Fisher Scientific) (40×). Scale: 75 µM.

Regarding the results of the SytoxGreen staining, all the treatments resulted in an increase in plasmatic membrane permeabilization (Figure 5), thus discarding the activation of a necrotic process after treatments with the five compounds.

## 3. Discussion

Three types of cell death have been described in trypanosomatids: apoptosis-like, necrosis, and autophagy. Apoptosis (or PCD) is a highly regulated process essential for different biological processes, the key events of which we mentioned in the introduction. Necrosis is another cell death process that can be regulated or unregulated, but in which plasma membrane disruption is the main feature. Autophagy is a mechanism that occurs in response to cellular stress, in which the cell degrades its content, such as damaged proteins or organelles, to be recycled [17]. In *Leishmania*, metacyclogenesis is dependent on autophagy, but if exacerbated, this autophagy can cause cell death. Stress conditions, such as treatment with different drugs, can induce an autophagic phenotype in trypanosomatids [18].

ROS generation and mitochondria also play an essential role in the PCD phenotype in unicellular eukaryotes. Furthermore, the imbalance of Ca2^+^ influx in the mitochondrion triggers oxidative stress in trypanosomatids, which leads to an apoptotic-like cell death phenotype [19].

It is also important to mention that another key factor in cell death fate is the energy/ATP level status. If a process of ATP starvation were to occur, the parasite would cease its metabolic activities, such as PCD, to carry out autophagy; normally, however, the autophagy process is used by trypanosomatids to carry out metacyclogenesis [20]. Moreover, the presence of this alternative death pathway, as well as the interaction between more than one cell death process, cannot be ruled out in these protozoa [18].

Taken together, compounds 3 and 5 seem to cause ROS accumulation, being the first effect of the compounds. This accumulation, which may be due to the calcium imbalance, probably also affects the mitochondrial membrane’s potential and, consequently, the ATP levels of the parasite, as we can see in the results. This event of mitochondrial dysfunction must trigger other mechanisms such as phosphatidylserine exposure, which is hallmark of classic apoptosis, confirming the apoptotic-like cell death in n *L. amazonensis* promastigotes upon the selected molecules separately. There are many examples in previous research of different natural or synthetic substances that are able to cause apoptosis in *Leishmania* parasites as the present compounds, including whitanolides, phenalenones, or oxasqualenoids [21,22,23,24]. On the other hand, compounds 7, 9, and 10 could trigger an autophagic process, due to their accumulation of ROS, without altering mitochondrial functions (i.e., their membrane potential and ATP levels); a necrotic process can be ruled out, since the plasmatic membrane of the parasite remains intact after treatment with the compounds. This is corroborated by the SytoxGreen assay [18].

In analyzing the group of structures each molecule belongs to (Table 4), we found that five featured a pyrimidin structure, indicating that this kind of benzene skeleton with two nitrogens replacing carbons plays important role in the biological activity. In addition, five of the compounds contained azoles, a class of five-membered heterocyclic compounds containing at least one nitrogen replacing carbons, which are a commonly known antifungal structures. Four of the selected compounds presented sulfonyl and sulfonamide groups, which is consistent with the data obtained in this study, since a large number of important drugs contain the sulfonamide group [25]. However, one interesting fact is that three of them feature a carboxamide in their structure, which is a relatively low reactive group, very resistant to hydrolysis, and present in many commercialized oral drugs.

Three of the eleven compounds were found to feature anti-leishmanial activity. Firstly, compound 9, a malarial inhibitor, was reported to feature activity against *L. donovani* promastigotes [26], followed by compound 5 [27], which belongs to kinetoplastids inhibitors, and compound 10, a tuberculosis inhibitor, that was reported to feature activity against intracellular amastigotes of the same species of *L. donovani* [28]. In addition, compounds 5, 7, 9, and 10 were previously reported to feature activity against another kinetoplastid organism, the causative agent of sleeping sickness disease, *Trypanosoma brucei brucei* trypomastigotes [27,28]; compound 9 was also found to be active against *T. evansi* trypomastigotes, the causative agent of an animal disease known as surra [29], and compound 10 was shown to be active against *T. cruzi* intracellular amastigotes, the causative agent of Chagas disease, as well as against *Plasmodium falciparum*, the causative agent of Malaria disease [28].

In relation to the possible targets of the molecules, just three of them were already described to inhibit or react with different eukaryotic proteins. The first example is compound 10, a well-known inhibitor of methionine aminopeptidase [27]; the second is compound 9, which inhibits NADPH oxidase 4 [30]; and the last one is compound 3, which interferes with thioredoxin reductase [31]. Regarding methionine aminopeptidase, the co-translational processing of N-terminal methionine is a highly evolutionarily conserved process; it is essential for the survival and proliferation of both prokaryotes and eukaryotes [32]. Cases of NADPH oxidase complex have not been identified in *Leishmania* genus, but it is supposed to feature ferric iron reductase as an analogue, which is thought to be the enzyme from which NADPH oxidases have evolved, because they share the same core domain and are very similar in structure [33]. Thioredoxin reductase performs multiple functions related to oxidoreductase in higher eukaryotes. In trypanosomatids this enzyme is very specific, it is called triparedoxin, and manages to perform the same functions [34], besides being a well-recognized specific target for new trypanocidal agents [35].

In relation to the ADME properties, we collected the data from human cytochrome inhibition and glutathione reactivity (Table 4), since we had already deleted the molecules with high cytotoxicity against eukaryotic cells. Relative to the cytochrome P450 inhibition activity, once we collected the data from the inhibition of two cytochromes, we observed that all compounds displayed IC_50_ values lower than 20 μM against one of the cytochrome, CYP2C9, which was important not just for the normal function of the enzyme but also in interactions with another drugs. This is because a depletion in the activity of CYP2C9 can affects the metabolism or elimination rate of another treatment causing drug–drug interaction [36]. Concerning the glutathione reactivity, and recalling that glutathione is the principal antioxidant of the cells, compounds 3, 5, and 6 exhibited medium levels of glutathione reactivity, representing a moderate risk to cells in recovering from oxidative stress [37].

## 4. Materials and Methods

### 4.1. Chemicals

The molecules were provided diluted in DMSO at 10mM and were stored at −20 °C. The full data on the Pathogen Box compounds are available at https://www.pathogenbox.org (accessed on 30 May 2021). A LCT-Premier XE TOF-MS (Waters, Milford, MA, USA) was employed for registering MS spectra in the 100–1500 m/z range of the selected molecules.

### 4.2. Strains

For the experiments, promastigotes of *Leishmania amazonensis* strain (MHOM/BR/77/LTB0016) were used; they were maintained in RPMI 1640 medium (Gibco, Massachusetts, USA) at 26 °C. Murine macrophages J774A.1 (ATCC #TIB-67), maintained in DMEM medium at 37 °C with 5% CO_2_ were used for the cytotoxic activity assays.

### 4.3. Leishmanicidal Activity

The screening of the 400 compounds against *L. amazonensis* promastigotes was initially tested at 10 µM, previously prepared according to the MMV foundation instructions provided with the box. The activity of the compounds was determined by using the modified alamarBlue^®^ assay (Invitrogen/Life Technologies, Madrid, Spain), as previously described [38], with 10^6^ promastigotes per well. The concentration of DMSO never exceeded 0.1% (*v*/*v*), avoiding the effect on the parasite’s proliferation or morphology. Subsequently, the plates were analyzed on an EnSpire multimode plate reader (PerkinElmer, MA, USA) to measure their fluorescence after 72 h. In addition, the compounds that exhibited a percentage of inhibition higher than the 90% were studied to elucidate their IC_50_ (inhibitory concentration 50). To this end, serial dilutions were performed in 96 well plates, and incubated with the parasites for 72 h after adding alamarBlue^®^. Next, the plates were analyzed on the EnSpire plate reader and the IC_50_ values were calculated by using SigmaPlot 14.0 (Systat Software Inc., Chicago, IL, USA). Each concentration was tested in duplicate and in three independent experiments for all the compounds.

The intracellular amastigote activity was measured by parasite rescue and transformation assay, also performed according to Jain et al. [39], for the most suited compounds (the group that exhibited an inhibition against the parasite higher than 91% at 10 µM). The macrophages were placed in a 96 well plate at a density of 2 × 10^5^/mL in DMEM and incubated for 5 h. Next, 1:10 of stationary phase promastigotes (7 day old culture) were added to the macrophages and re-incubated at 37 °C and 5% of CO_2_ overnight to allow the parasites to infect the cells. After the incubation, the excess promastigotes were washed off and serial dilutions of the compounds were added. After 24 h of incubation, we removed the medium and added 30 μL of Schneider medium with 0.05% SDS to break the macrophages. The plate was shaken for 30 s and a further 180 μL of medium was added to each well. The plates were then incubated at 26 °C for 72 h for the transformation of the rescued amastigote into promastigotes. AlamarBlue^®^ was added and the plates were measured and analyzed as discussed above.

### 4.4. Cytotoxic Activity

The cytotoxic effect of the selected molecules was calculated following the same protocol to that previously mentioned in the leishmanicidal activity section. On this occasion, murine macrophages were the cell line selected to perform this biological activity. First, the macrophages were seeded on the plates, serial dilutions of the compound were added and, finally, the alamarBlue^®^ was added at 10%. The plates were incubated for 24 h before measurement, and the CC_50_ values were calculated as described above. As in the previous assay, each concentration was tested in duplicate and in three independent experiments for each compound.

### 4.5. ATP Levels

To detect alterations due to the different treatments on the cellular ATP levels, CellTiter-Glo (Promega) was utilized. Briefly, the promastigotes were incubated with the IC_90_ of the compounds for 24 h at 26 °C, then harvested and incubated with the kit, gently mixed for two minutes, and incubated for a further 10 min. Finally, luminescence was measured on the EnSpire spectrophotometer (Perkin Elmer) [40]. The experiment was performed at least three times in duplicate on different days.

### 4.6. Mitochondrial Membrane Potential Disruption

For this proposal, the JC-1 Mitochondrial Membrane Potential kit (Cayman Chemical) was used following the manufacturer’s instructions, which consisted in incubating the parasites with the IC_90_ of the compounds for 24 h in order to subsequently wash the cells and incubate with the reagents of the kit (buffer and JC-1 dye). A total of three independent experiments were conducted for this assay. The JC-1 acts as a monomer or dimer depending on the mitochondrial membrane potential; at low potential, it remains a monomer, which emits green fluorescence, while in normal conditions it remains as a dimer, which emits fluorescence in red, and gets stuck inside mitochondria [41].

### 4.7. Reactive Oxygen Species (ROS) Detection

CellRox DeepRed (Invitrogen) fluorescent dye was used for the determination of the ROS accumulation. The protocol consisted in incubating the parasites with the IC_90_ of the selected molecules for 24 h, and then washing them and adding the CellRox at 5 µM. After 30 min in darkness, the cells were observed under a fluorescent inverted microscope EVOS FL (Life Technologies, Thermo Fisher Scientific, Waltham, MA, USA). This staining was performed three times in independent tests. A positive control was performed as the internal control and corresponded to cells in which ROS production was achieved through the incubation of parasites with 600 µM hydrogen peroxide 30 min before detection [42].

### 4.8. Phosphatidylserine Externalization

To measure the level of population that experiments phosphatidylserine externalization from the inner to the outer part of the cytoplasmic membrane, an Apoptosis kit for Tali (Invitrogen, Thermo Fisher Scientific, Waltham, MA, USA) was utilized. The kit employs annexin-v, which binds to the external phosphatidylserine, and propidium iodide, that penetrates dead cells, to classify the population of the cells as: alive (any stain), dead (propidium stained), and apoptotic (annexin stained). After incubating the parasites for 24 h with the IC_90_ of the compounds, the cells were washed and incubated with the kit by following the instructions. A minimum of three independent experiments were performed for this assay. The cells were then quantified by the image-based cytometer Tali^®^ (Life Technologies, Thermo Fisher Scientific, Waltham, MA, USA) [43].

### 4.9. Plasmatic Membrane Permeability

In order to detect membrane permeability alterations, the SYTOX^®^ Green assay was performed on the parasites. Briefly, the promastigotes were incubated with the IC_90_ of the compounds and incubated 24 h at 26 °C. SYTOX^®^ Green was added at a final concentration of 1 μM (Molecular Probes^®^, Thermo Fisher Scientific, Waltham, MA, USA) for 30 min in the dark. Subsequently, the protozoa were disposed to black plates and the fluorescence was measured using an EnSpire^®^ Multimode Plate Reader (Perkin Elmer, Madrid, Spain) with an excitation wavelength of 504 nm and an emission wavelength at 523 nm. The experiment was performed at least three times on different days to corroborate the results. The increase in fluorescence was correlated with the binding of the dye with the DNA of the promastigote. A positive control was carried out as the internal control, corresponding to the full permeabilization of the cells achieved by the addition of 0.1% Triton X-100 [44].

### 4.10. Statistical Analyses

The data are presented as the mean ± standard deviation (SD) from at least three independent experiments, and the data shown are the representative results. The inhibitory concentrations (IC_50_ and CC_50_) were calculated by non-linear regression analysis with 95% confidence limits. Statistical differences between means were tested using a one-way analysis of variance (ANOVA; three or more samples), with a post-hoc pairwise comparisons of means carried out using Tukeyʼs test, using the SigmaPlot 12.0 software. A significance level of *p* < 0.05 was used.

## 5. Conclusions

The present study identified ten hit compounds that offer desirable inhibitory activity against the promastigotes of *L. amazonensis.* MMV676388 (**3**), MMV690103 (**5**), MMV022029 (**7**), MMV022478 (**9**) and MMV021013 (**10**), were the five molecules that stood out for their favorable properties and which correspond to the structures of: one tetrazole, three pyrimidines and one sulfonamide; and induced a mechanism of controlled cell death without instigating an undesirable immune response. The efficacy and safety of the drugs in vitro do not necessarily reflect the situation in vivo, as pharmacokinetic factors of the compound also exert an influence, but they are quite close to reality. However, an in-depth investigation would help to improve the knowledge of their mechanism of action against *L. amazonensis*; there is also to the possibility of exploring and finding new leishmanicidal agents in the aforementioned structures.

## Figures and Tables

**Table 1:** Leishmanicidal activity against promastigote stage of the best compounds from MMV Pathogen Box, and cytotoxic effect against murine macrophages (µM). Selectivity index (CC_50_/IC_50_).

Compound	*Leishmania amazonensis* Promastigotes IC_50_ (µM)	Macrophages CC_50_ (µM)	Selectivity Index
1	6.43 ± 1.06	>10	>1.56
2	4.03 ± 0.73	>10	>2.48
3	1.33 ± 0.12	8.02 ± 0.93	5.66
4	1.44 ± 0.24	4.30 ± 0.16	2.94
5	0.59 ± 0.08	>10	>17.06
6	3.93 ± 0.64	>10	>2.54
7	1.84 ± 0.14	>10	>5.44
8	2.62 ± 0.40	8.01 ± 1.13	3.06
9	0.77 ± 0.01	7.22 ± 2.36	9.35
10	1.85 ± 0.39	>10	>5.40
Miltefosine	6.48 ± 0.24	72.19 ± 3.06	11.14

**Table 2:** 2D Chemical structures of the best active compounds.

Comp. 1	Comp. 2
** 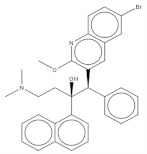 **	** 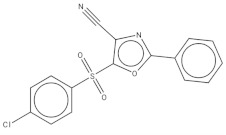 **
Comp. 3	Comp. 4
** 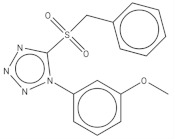 **	** 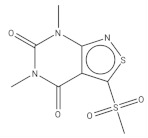 **
Comp. 5	Comp. 6
** 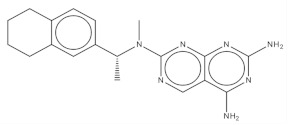 **	** 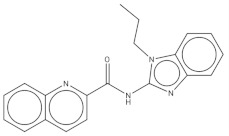 **
Comp. 7	Comp. 8
** 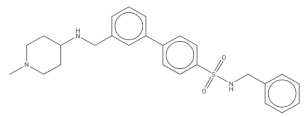 **	** 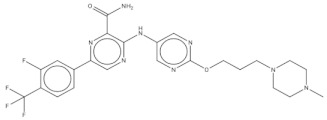 **
Comp. 9	Comp. 10
** 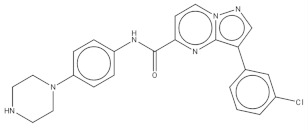 **	** 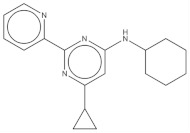 **

**Table 3:** Leishmanicidal activity against intracellular amastigote stage of the best compounds from MMV Pathogen Box (µM). Selectivity index (CC_50_/IC_50_). nd: not determined.

Compound	*Leishmania amazonensis* Intracellular Amastigote IC_50_ (µM)	Selectivity Index
1	>10	nd
2	>10	nd
3	4.78 ± 1.20	1.68
4	>10	nd
5	1.25 ± 0.28	>8.00
6	8.52 ± 0.67	>1.17
7	2.50 ± 0.27	>4.00
8	6.85 ± 0.75	1.17
9	6.90 ± 1.79	1.05
10	1.33 ± 0.36	>7.52
Miltefosine	3.12 ± 0.30	23.14

**Table 4:** Hit molecules and their characteristics. ^a^: Data from mmv.org (accessed on 15 May 2021).

MMV Identifier ^a^	CHEMBL Identifier and Name	ADME ^a^		
		CYP2C9 IC_50_ (μM)	CYP2D6 IC_50_ (μM)	Glutathione Reactivity ^a^
MMV676388 (Compound 3)	CHEMBL3637869 5-(Benzylsulfonyl)-1-(3-methoxyphenyl)-1H-tetrazole	>20	15	Medium
MMV690103 (Compound 5)	CHEMBL3637900 2-N-Methyl-2-N-[(1R)-1-(5,6,7,8-tetrahydronaphthalen-2-yl)ethyl]pyrimido [4,5-d]pyrimidine-2,5,7-triamine	>20	>20	Medium
MMV022029 (Compound 7)	CHEMBL545853 N-Benzyl-4-[3-[[(1-methylpiperidin-4-yl)amino]methyl]phenyl]benzenesulfonamide	>20	>20	No GSH adduct
MMV022478 (Compound 9)	CHEMBL534797 3-(3-Chlorophenyl)-N-(4-piperazin-1-ylphenyl)pyrazolo [1,5-a]pyrimidine-5-carboxamide	>20	>20	No GSH adduct
MMV021013 (Compound 10)	CHEMBL530275 N-Cyclohexyl-6-cyclopropyl-2-pyridin-2-ylpyrimidin-4-amine	>20	>20	No GSH adduct

## Data Availability

Data is contained within the article and Supplementary Materials.

## Supplementary Materials

The following are available online at https://www.mdpi.com/article/10.3390/ph14121219/s1, Table S1: Molecular information of the 57 compounds that presents leishmanicidal activity; Scheme S1: Concentration-dependent curves for the IC50 values of the 11 active compounds; Table S2: Information about the Order and batch of the Pathogen Box plates; Scheme S2: MS spectra of the selected compounds.
