# Supplementary material for: Discovery of New Chemical Tools against Leishmania amazonensis via the MMV Pathogen Box
Source: Pharmaceuticals (Basel). 2021 Nov 24;14(12):1219. doi: 10.3390/ph14121219 (PMC8708704; doi:10.3390/ph14121219)
Supplement: Supplementary file 1 [file pharmaceuticals-14-01219-s001.zip › pharmaceuticals-1347528-supplementary.pdf]

**Table S1.** Molecular information of the 57 compounds that presents leishmanicidal activity.

| MMV Identifier | Inhibitor | % of inhibition[a] | Mol. structure | MMV Identifier | Inhibitor | % of inhibition[a] | Mol. structure |
|---|---|---|---|---|---|---|---|
| MMV689758 | R | 90 | 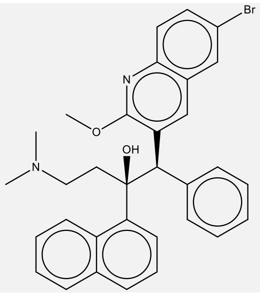 | MMV1030799 | M | 91 | 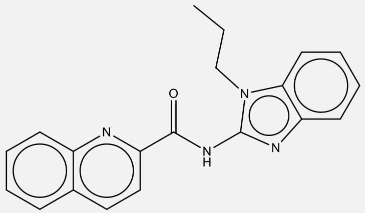 |
| MMV676412 | T | 93 | 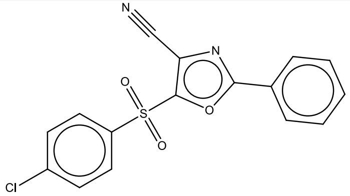 | MMV690102 | K | 89 | 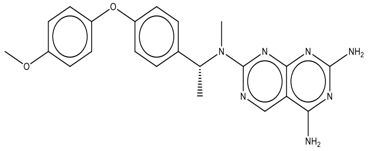 |
| MMV676401 | T | 84 | 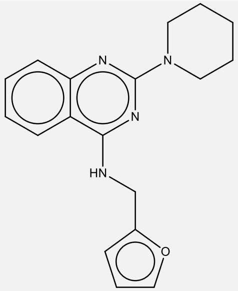 | MMV688179 | K | 86 | 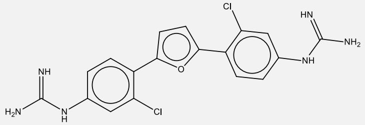 |

| | | | | | | | |
|---|---|---|---|---|---|---|---|
| MMV676477 | T | 92 | 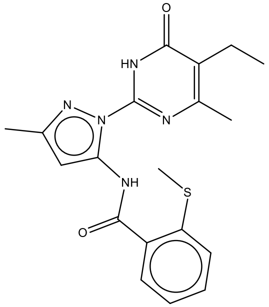 | MMV688262 | T | 86 | 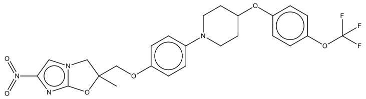 |
| MMV661713 | T | 84 | 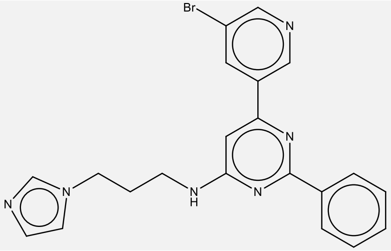 | MMV687807 | T | 90 | 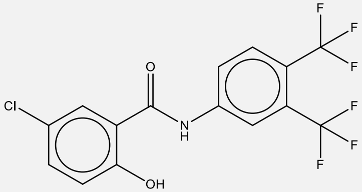 |
| MMV676383 | T | 83 | 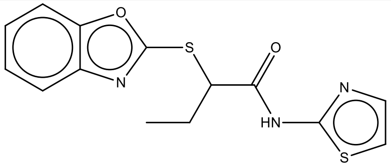 | MMV021660 | T | 87 | 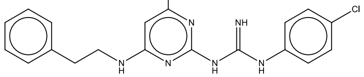 |
| MMV676388 | T | 91 | 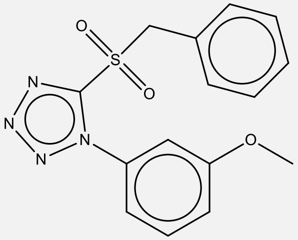 | MMV688417 | X | 87 | 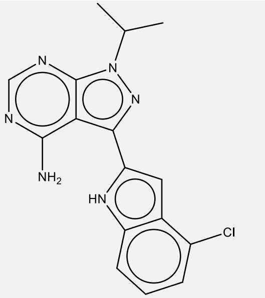 |

| | | | | | | | |
|---|---|---|---|---|---|---|---|
| MMV688936 | T | 90 | 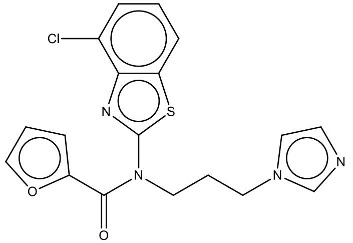 | MMV687273 | T | 89 | 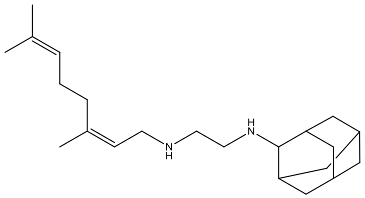 |
| MMV676509 | T | 80 | 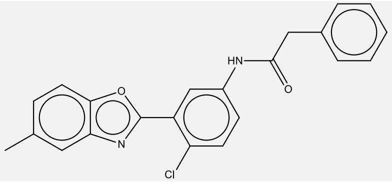 | MMV1088520 | M | 89 | 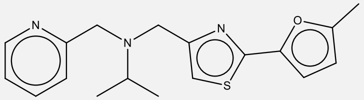 |
| MMV461553 | T | 88 | 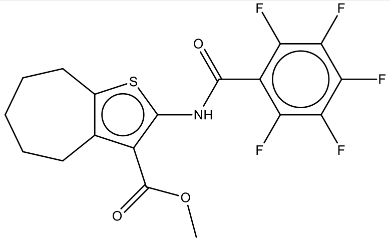 | MMV688844 | T | 80 | 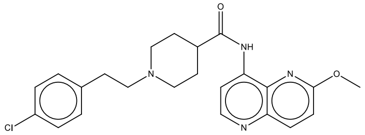 |
| MMV000062 | R | 89 | 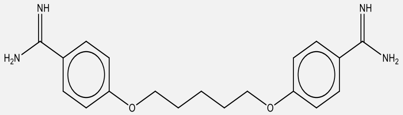 | MMV689437 | K | 89 | 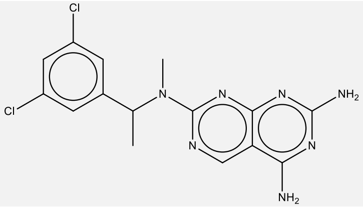 |
| MMV011903 | M | 86 | 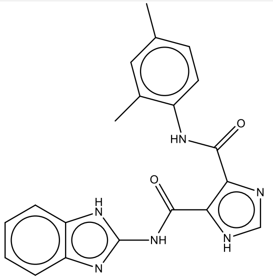 | MMV022029 | M | 91 | 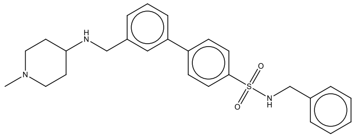 |

| | | | | | | | |
|---|---|---|---|---|---|---|---|
| MMV688761 | S | 88 | 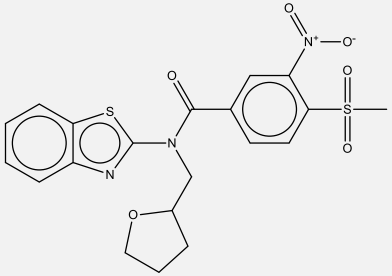 | MMV024114 | M | 92 | 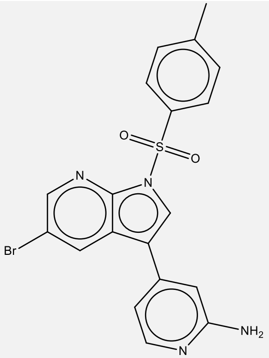 |
| MMV676604 | K | 66 | 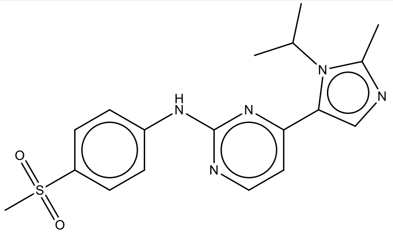 | MMV024406 | M | 92 | 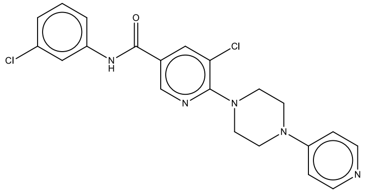 |
| MMV687776 | L | 52 | 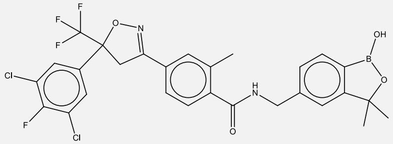 | MMV659004 | K | 87 | 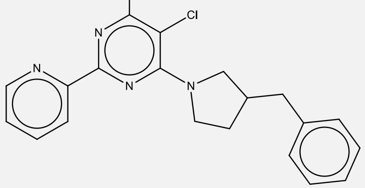 |
| MMV687800 | R | 88 | 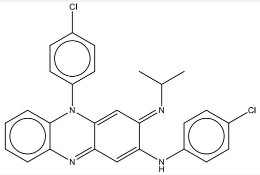 | MMV687812 | T | 91 | 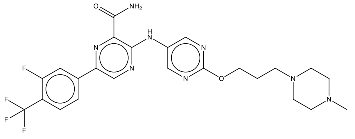 |
| MMV676605 | M | 53 | 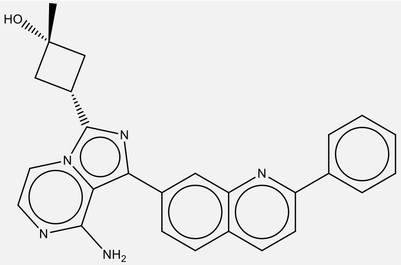 | MMV688372 | K | 87 | 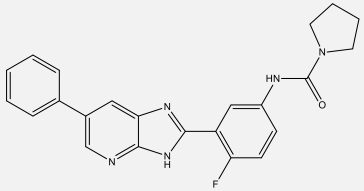 |

| | | | | | | | |
|---|---|---|---|---|---|---|---|
| MMV688763 | S | 86 | 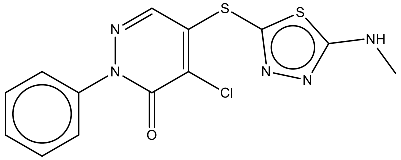 | MMV022478 | M | 91 | 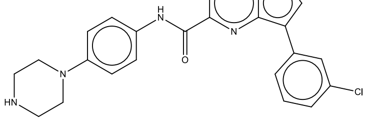 |
| MMV637229 | H | 61 | 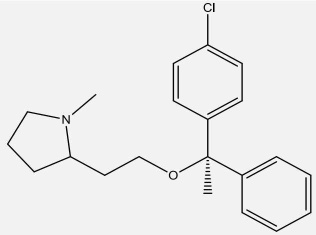 | MMV675968 | C | 91 | 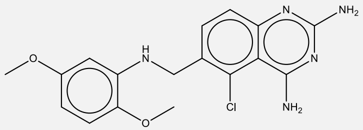 |
| MMV689480 | R | 87 | 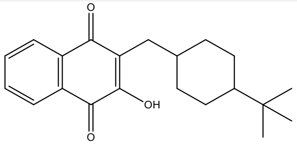 | MMV688938 | T | 85 | 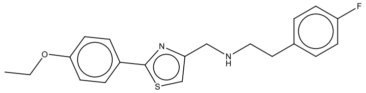 |
| MMV688768 | S | 86 | 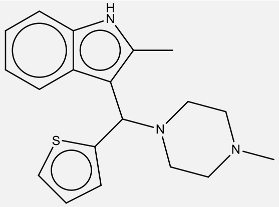 | MMV671636 | O | 66 | 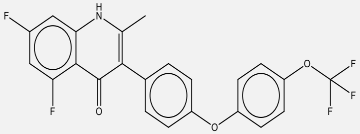 |
| MMV000016 | R | 86 | 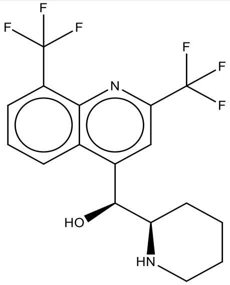 | MMV687765 | T | 82 | 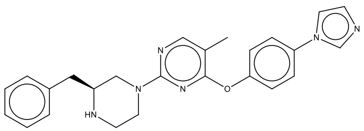 |

| | | | | | | | |
|---|---|---|---|---|---|---|---|
| MMV006901 | M | 85 | 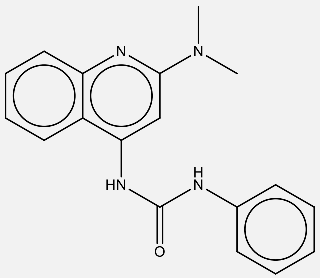 | MMV153413 | T | 86 | 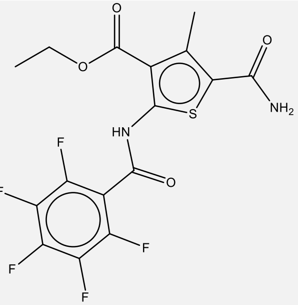 |
| MMV687730 | T | 87 | 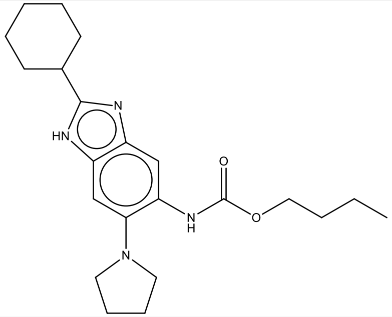 | MMV272144 | T | 89 | 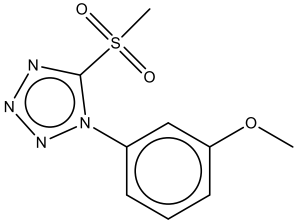 |
| MMV688371 | K | 89 | 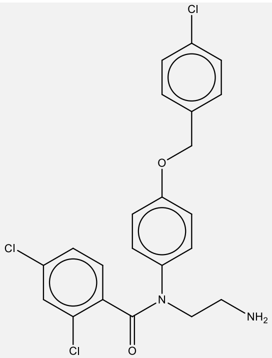 | MMV021013 | T | 90 | 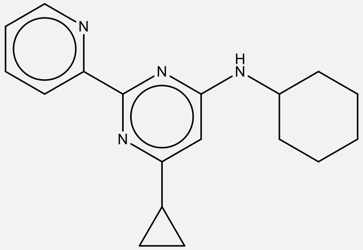 |

| | | | | | | | |
|---|---|---|---|---|---|---|---|
| MMV688283 | K | 85 | 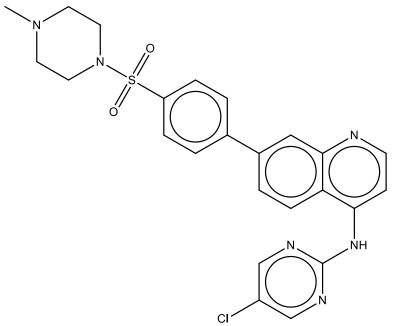 | MMV688754 | K | 76 | 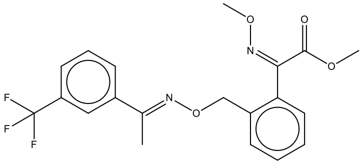 |
| MMV687703 | T | 85 | 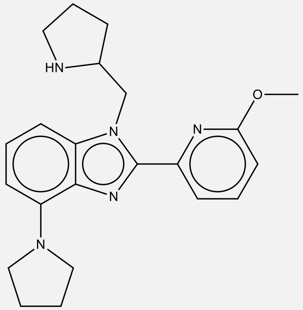 | MMV688978 | R | 90 | 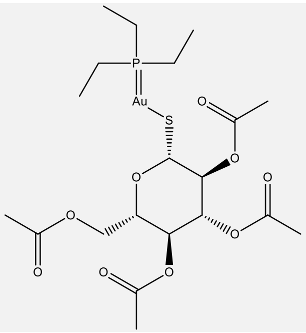 |
| MMV687251 | T | 91 | 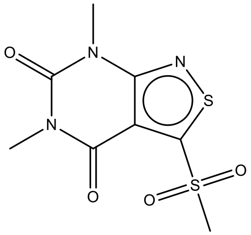 | MMV688273 | K | 79 | 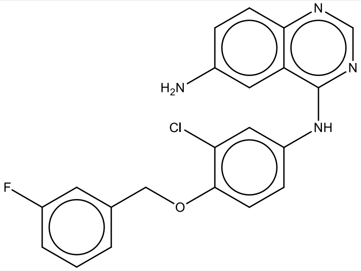 |
| MMV690103 | K | 90 | 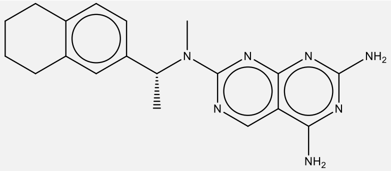 | | | | |

R: Reference compound; T: Tuberculosis; M: Malaria; K: Kinetoplastid; X: Toxoplasmosis; C: Cryptosporidiosis; O: Onchocerciasis; S: Schistosomiasis; L: Lymphatic filariasis; H: Hookworm Trichuriasis. *% of inhibition at 10 μM (against *L. amazonensis* promastigotes)

| | |
|---|---|
| Comp. 1 | 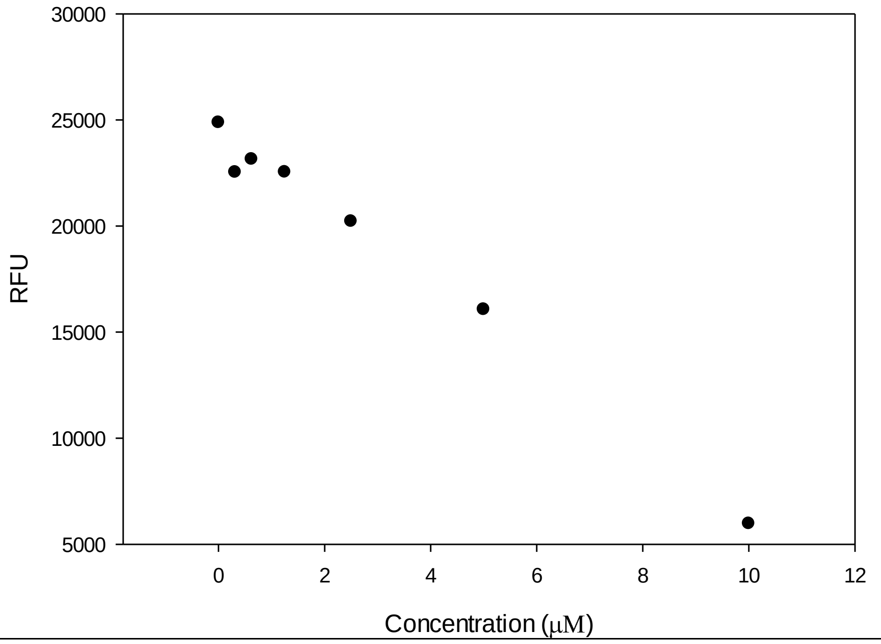30000<br>25000<br>20000<br>15000<br>10000<br>5000<br>RFU<br>0<br>2<br>4<br>6<br>8<br>10<br>12<br>Concentration (μM) |
| Comp. 2 | 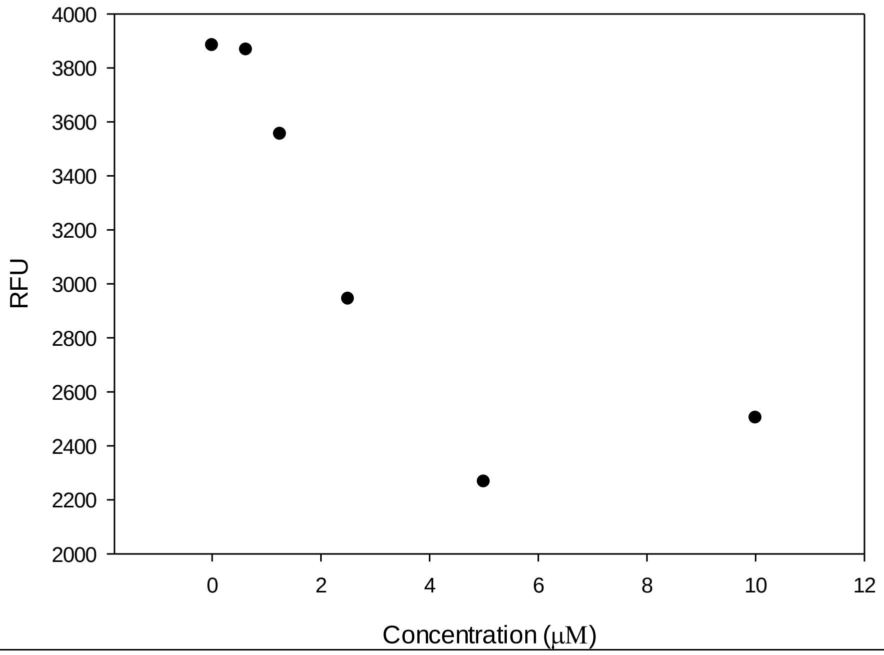4000<br>3800<br>3600<br>3400<br>3200<br>3000<br>2800<br>2600<br>2400<br>2200<br>2000<br>RFU<br>0<br>2<br>4<br>6<br>8<br>10<br>12<br>Concentration (μM) |

| | |
|---|---|
| Comp. 3 | 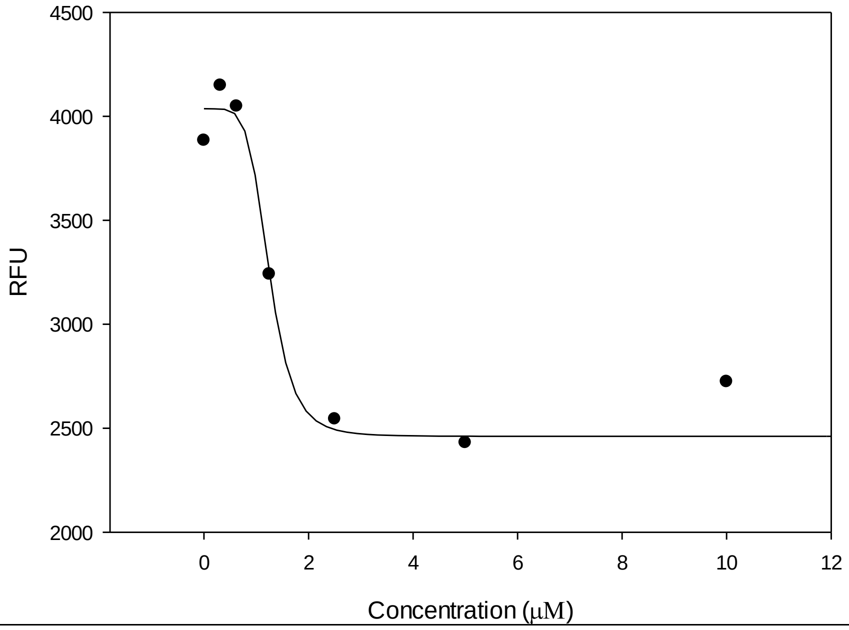RFU<br>Concentration (μM)<br>4500<br>4000<br>3500<br>3000<br>2500<br>2000<br>0<br>2<br>4<br>6<br>8<br>10<br>12 |
| Comp. 4 | 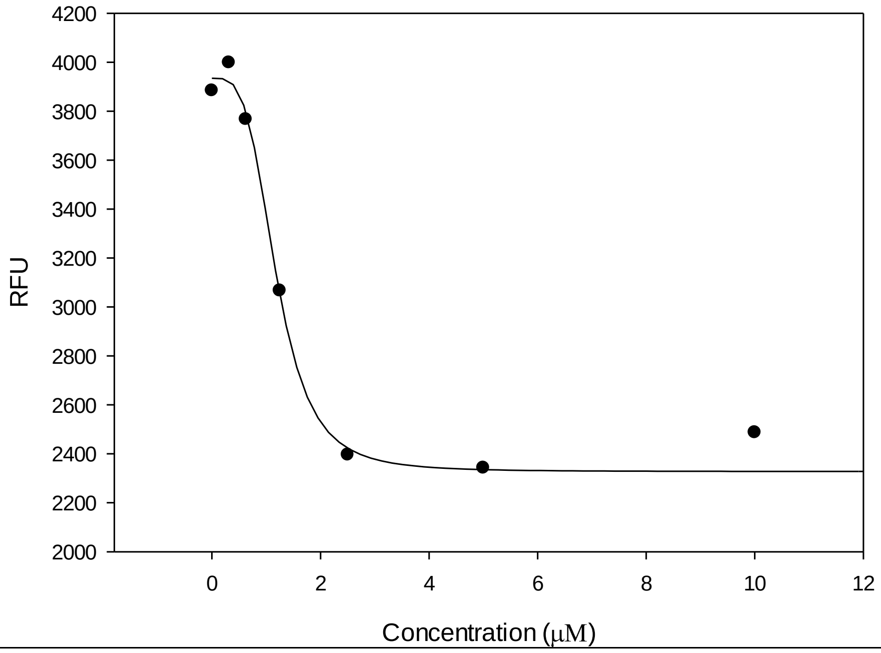RFU<br>Concentration (μM)<br>4200<br>4000<br>3800<br>3600<br>3400<br>3200<br>3000<br>2800<br>2600<br>2400<br>2200<br>2000<br>0<br>2<br>4<br>6<br>8<br>10<br>12 |

<table>
<tr><td>Comp. 5</td><td>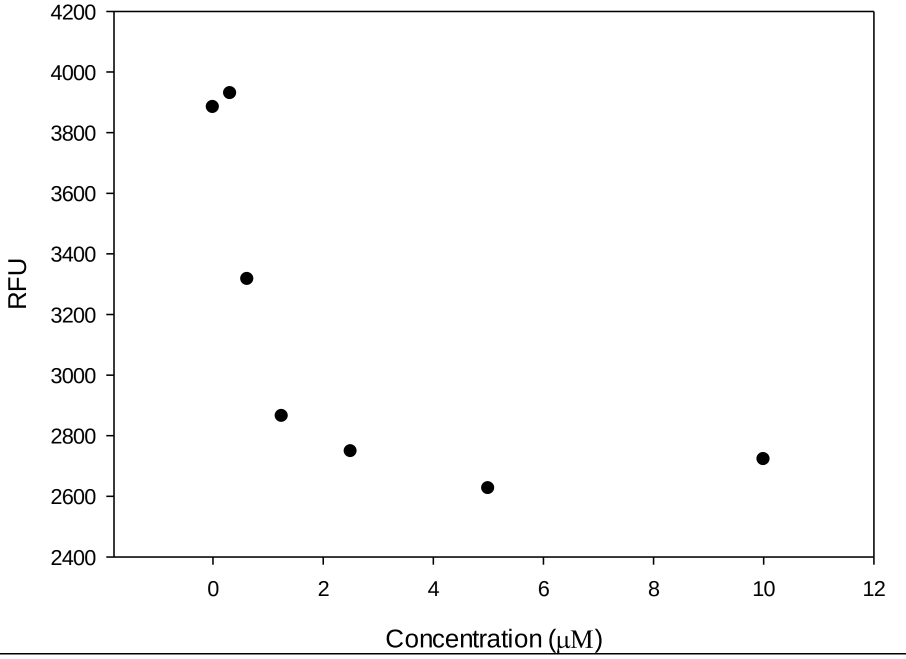
</td></tr>
<tr><td>Comp. 6</td><td>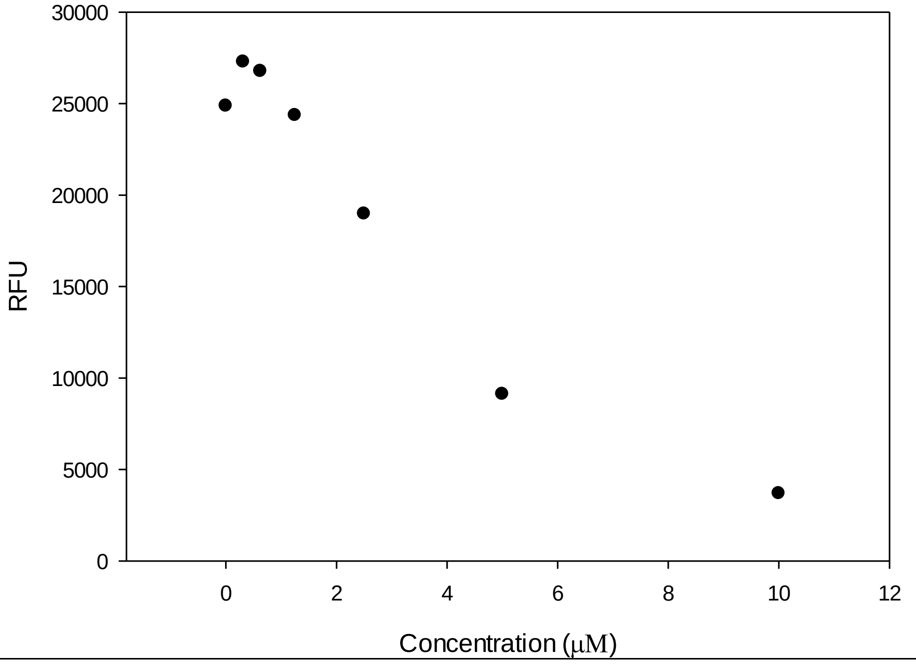
</td></tr>
</table>

<table>
<tr>
<td>Comp.<br>7</td>
<td>
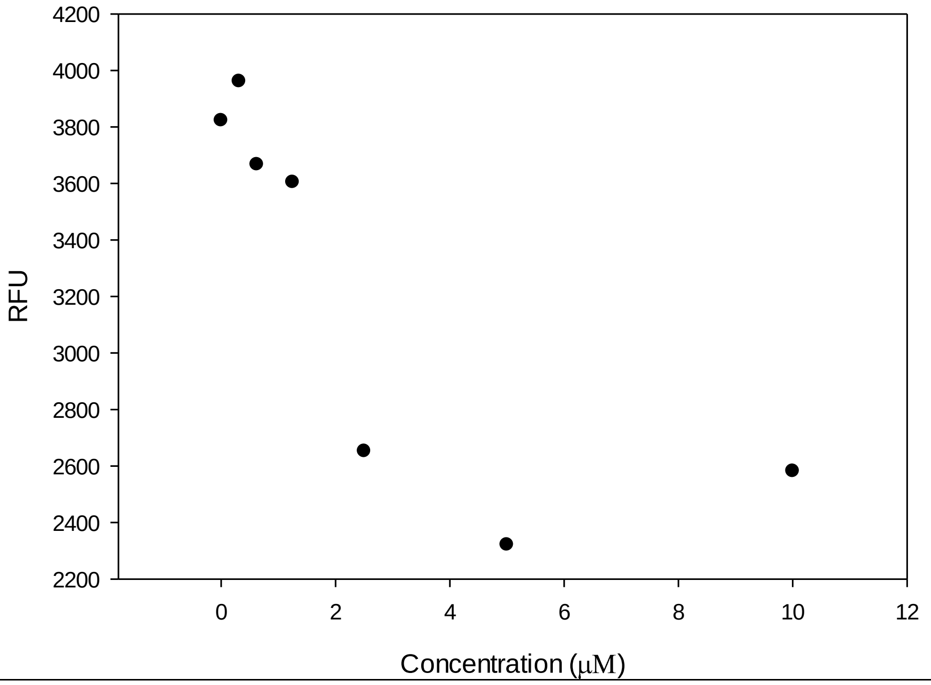

</td>
</tr>
<tr>
<td>Comp.<br>8</td>
<td>
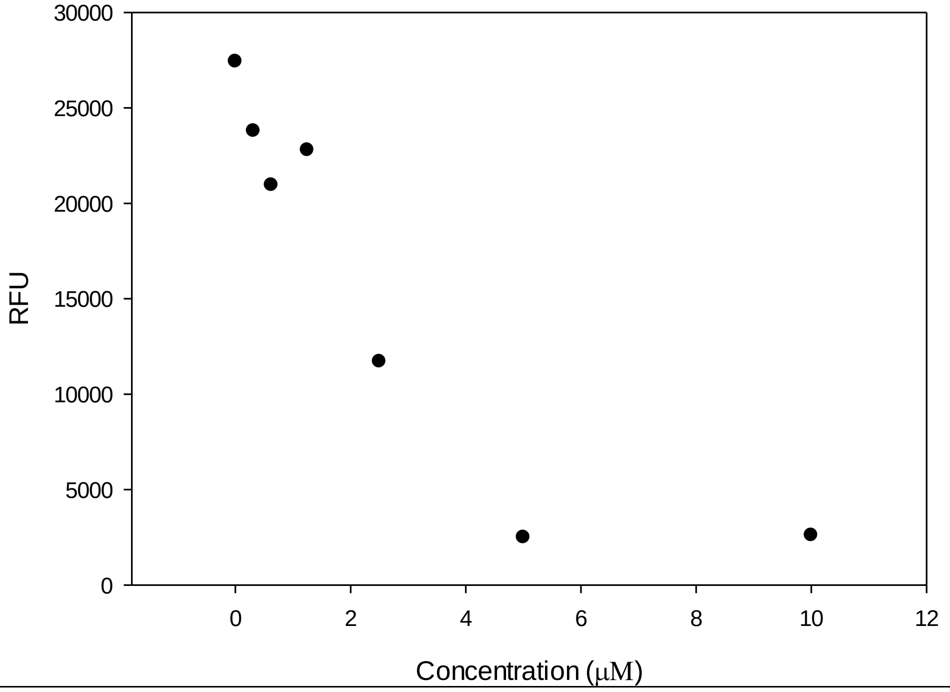

</td>
</tr>
</table>

Comp. 9

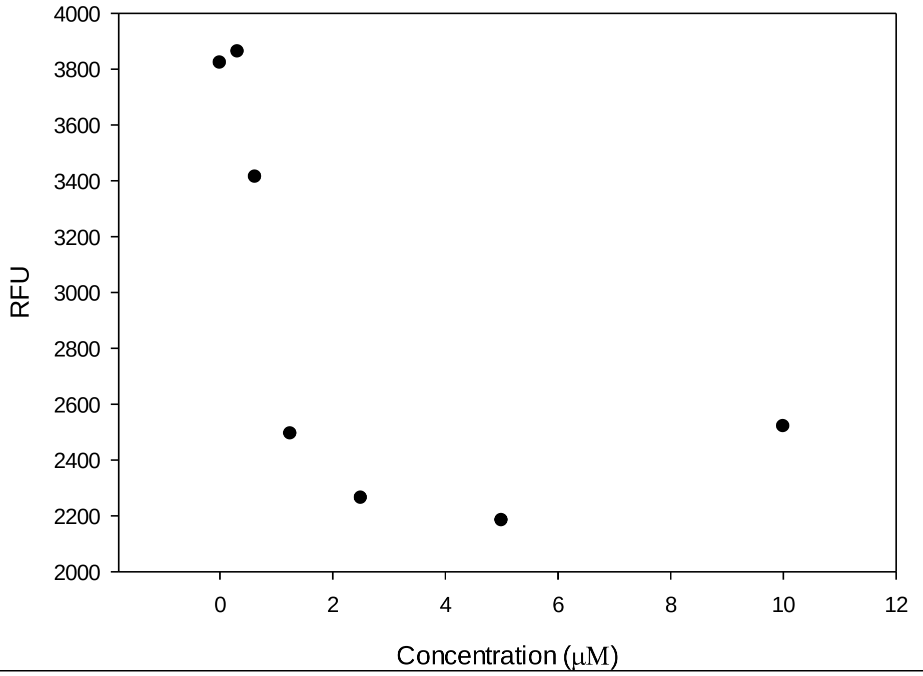


Comp. 10

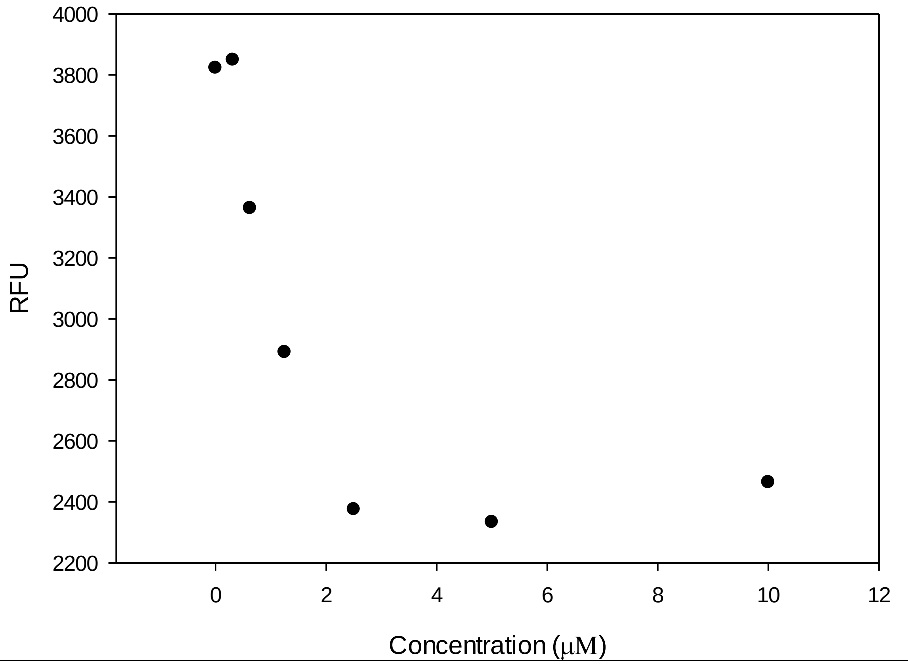


**Scheme S1.** Concentration-dependent curves for the $IC_{50}$ values of the 10 active compounds.

**Table S2.** Information about the Order and batch of the Pathogen Box plates. MMV- Pathogen Box - Order 1660792 (https://www.mmv.org/mmv-open/pathogen-box/pathogen-box-supporting-information, access date 15 May 2021).

| Plate | Batch |
|---|---|
| Plate A | BFD0014581 |
| Plate B | BFD0014681 |
| Plate C | BFD0014781 |
| Plate D | BFD0014881 |
| Plate E | BFD0014981 |

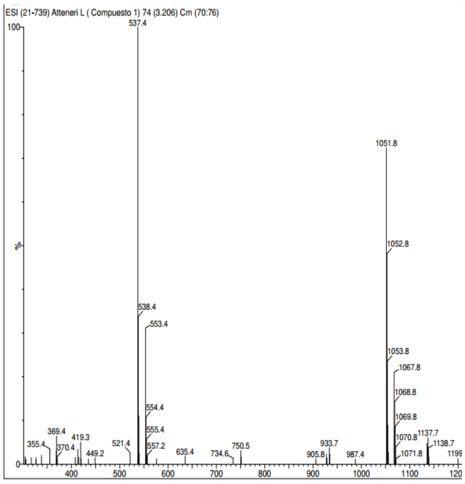


Compound 1

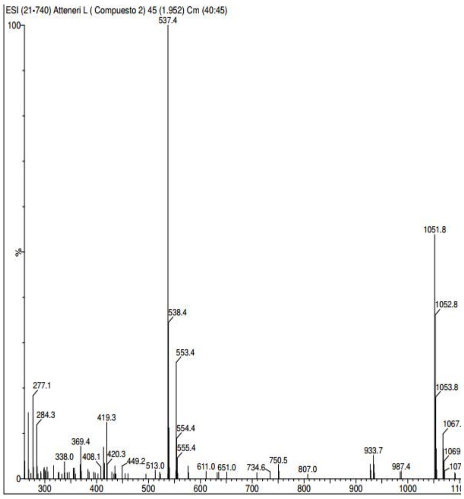


Compound 2

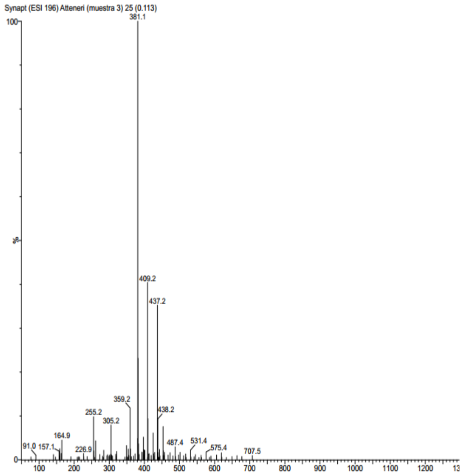


Compound 3

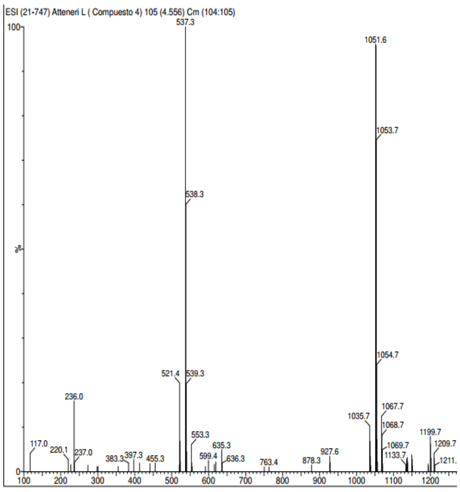


Compound 4

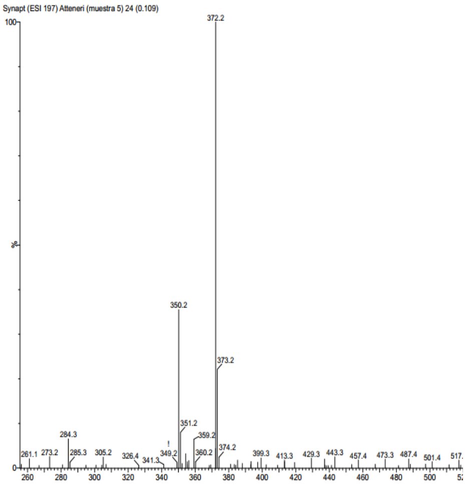


Compound 5

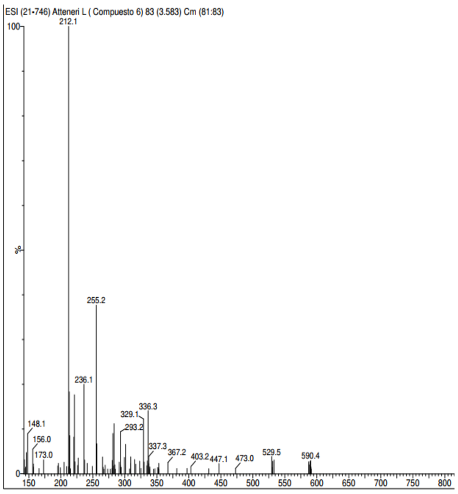


Compound 6

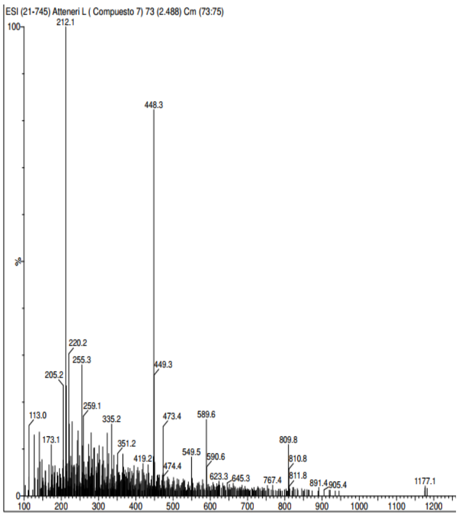


Compound 7

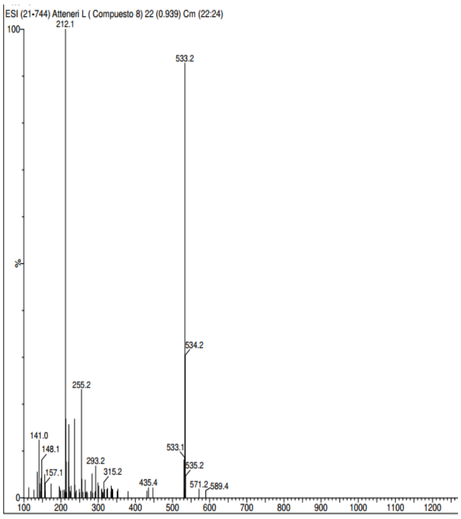


Compound 8

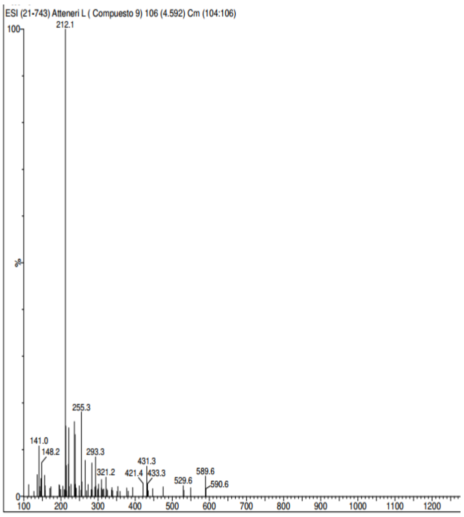


Compound 9

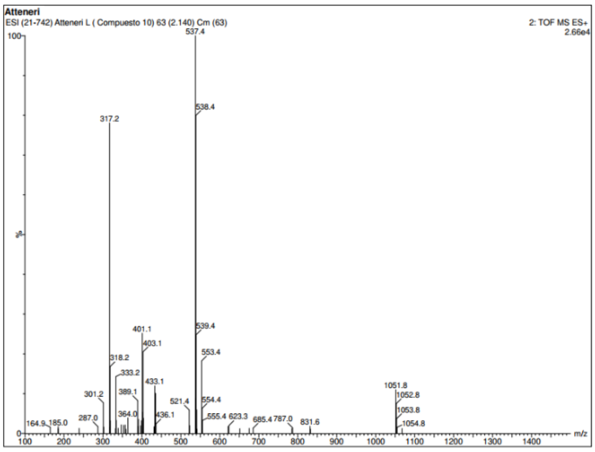


Compound 10

**Scheme S2.** MS spectra of the 10 selected compounds.
